# What can we experience and report on a rapidly presented image? Intersubjective measures of specificity of freely reported contents of consciousness

**DOI:** 10.12688/f1000research.75364.3

**Published:** 2024-08-23

**Authors:** Zhang Chuyin, Zhao Hui Koh, Regan Gallagher, Shinji Nishimoto, Naotsugu Tsuchiya

**Affiliations:** 1 Turner Institute for Brain and Mental Health & School of Psychological Sciences, Faculty of Medicine, Nursing, and Health Sciences, Monash University, Melbourne, Victoria, 3168, Australia; 2 Osaka University, Suita, Osaka, Japan; 3 Center for Information and Neural Networks (CiNet), National Institute of Information and Communications Technology, Suita, Osaka, Japan; 4 Advanced Telecommunications Research Computational Neuroscience Laboratories, Hikaridai, Kyoto, Japan

**Keywords:** Consciousness, free report, rapid scene, metacognition, confidence, gist, intersubjective agreement

## Abstract

**Background:** A majority of previous studies appear to support a view that human observers can only perceive coarse information from a natural scene image when it is presented rapidly (<100ms, masked). In these studies, participants were often forced to choose an answer from options that experimenters preselected. These options can underestimate what participants experience and can report on it. The current study aims to introduce a novel methodology to investigate how detailed information participants can report after briefly seeing a natural scene image.

**Methods:** We used a novel free-report paradigm to examine what people can freely report following a rapidly presented natural scene image (67/133/267ms, masked). N = 670 online participants typed up to five words to report what they saw in the image together with confidence of the respective responses. We developed a novel index, Intersubjective Agreement (IA). IA quantifies how specifically the response words were used to describe the target image, with a high value meaning the word is not often reported for other images. Importantly, IA eliminates the need for experimenters to preselect response options.

**Results:** The words with high IA values are often something detailed (e.g., a small object) in a particular image. With IA, unlike commonly believed, we demonstrated that participants reported highly specific and detailed aspects of the briefly (even at 67ms, masked) shown image. Further, IA is positively correlated with confidence, indicating metacognitive conscious access to the reported aspects of the image.

**Conclusion:** These new findings challenge the dominant view that the content of rapid scene experience is limited to global and coarse gist. Our novel paradigm opens a door to investigate various contents of consciousness with a free-report paradigm.

## Introduction

Intuitively, we have an impression that our visual experience is immensely rich. When we look at a complex natural scene, we can rapidly extract meaningful information and categorize it accurately in as short as 150 ms ( Li *et al.*, 2002; Biederman, 1981; Fabre-Thorpe *et al.*, 2001). In certain situations, the performance accuracy is correlated with confidence rating ( Fu *et al.*, 2016; Thunell & Thorpe, 2019), indicating some level of metacognitive monitoring is possible. Rapidly extracted information in this context is often called “scene-gist understanding” ( Oliva & Torralba, 2006) or “gist” in short. Previous studies demonstrate excellent human capability to rapidly categorise a natural scene based on the scene gist in a global and coarse manner. However, it is unclear whether we have conscious access to more detailed information upon seeing a complex natural image briefly ( Bayne & McClelland, 2018; McClelland & Bayne, 2016). The current study will focus on the information beyond “gist”, so that we will use a more general term: rapid scene experience.

In fact, the current dominant view on contents of rapid scene experience is that it is about high-level descriptions of the scene, which is coarse and lacks the fine details ( Campana & Tallon-Baudry, 2013; Fei-Fei *et al.*, 2007; Greene *et al.*, 2015; Kimchi, 1992; Oliva & Torralba, 2006; Greene & Fei-Fei, 2014).

This dominant view inherits the idea from the Gestalt theory of perception ( Koffka, 1922). One of the Gestalt principles states our tendency to perceive a scene as a whole rather than individual parts. This idea was further developed by Navon (1977) in his global precedent hypothesis. This hypothesis posits that visual perception first processes global features of an image, then proceeds to analyze local features. Supplemented by further experimental evidence, Campana and Tallon-Baudry (2013) recently extended this idea into conscious perception, where they claim conscious perception starts at the global and coarse level before the local and detailed level.

Taxonomy-based levels of categorization (“semantic categorization”; Rosch, 1998) was frequently used to study the content of rapid scene experience. The categories varied in hierarchies based on their degree of general-ness or specific-ness. In this categorization, the content of rapid scene experience can be reported either at the superordinate level or the basic level ( Fei-Fei *et al.*, 2007; Larson *et al.*, 2014; Walther *et al.*, 2009), but less likely at the subordinate level ( Malcolm *et al.*, 2012), with the subordinate level being the most specific category. Further evidence shows that the superordinate categorization is made prior to the basic-level categorization ( Loschky & Larson, 2010; Sun *et al.*, 2016). These findings provide evidence for the dominant view.

Overall, studies in this field often used forced-choice discrimination tasks, such as identifying the same image through a series of rapidly presented images (e.g., Thunell & Thorpe, 2019), ascertaining the superordinate category (natural-ness, urban-ness) of a scene (e.g., Loschky & Larson, 2010), and determining if the briefly viewed images contain animals (e.g., Thorpe *et al.*, 1996) or certain objects (e.g., Hollingworth, 1998). However, there are two methodological limitations in the forced-choice paradigm.

First, the forced-choice paradigm restricts the response options. The studies that employ the forced-choice paradigm often implicitly assume that participants can only process the images at a certain level (usually coarse). Due to the limited response options, participants were unable to report detailed information about their experience even if they perceived it ( Haun *et al.*, 2017). Researchers may employ the forced-choice paradigm just for the sake of convenience. However, other paradigms, such as free-report paradigms, are potentially more difficult to verify from a third-person view ( Dennett, 2007).

Second, the forced-choice paradigms create expectations of the content of images that will be presented for participants. For example, asking participants to choose between “animal” and “non-animal” will tell them they might see an animal. This could help participants perceive more detailed information ( McLean *et al.*, 2021; Sun *et al.*, 2017), while it can suppress seeing other aspects of rapidly presented scenes.

To overcome these problems, here we introduce a novel free-report paradigm. First, it addresses the restrictions on what participants can report. They freely reported what they saw in a briefly presented image and rated their confidence in the report. Second, to reduce any expectation that participants might have about the upcoming image category, we used a wide array of 412 natural and 8 artificial images and did not tell participants any information about the types of the images that they will see or types of the responses that we expect from them.

Of course, these modifications generate new challenges: how can we scientifically investigate such unconstrained data? What can we do to eliminate bias from us, the experimenters, on what participants should report? Rather than manually going through each image to define the correct answers, we utilize the majority vote of the participants. In other words, based on a large baseline of what participants report, we quantify the degree of specificity of reported words, which we call “intersubjective agreement (IA)”. For this purpose, we recruited a large pool of 600 participants. With our novel index applied to the free-report paradigm, we reexamine the dominant view that the content of rapid scene experience is limited to coarse information lacking in detail. The current study will answer if people can consciously see and report the details of an unexpected image at a brief glance.

## Results

To summarise, our novel index quantifies how “specific” a response is, with respect to a certain image. IA ranges from 0 to 1. A high IA means the word is specifically reported under the target image and not reported under other images, signifying detailed information. A low IA (~0.5) means the word is reported nonspecifically for all the images, signifying coarse information.

**Figure 1.**
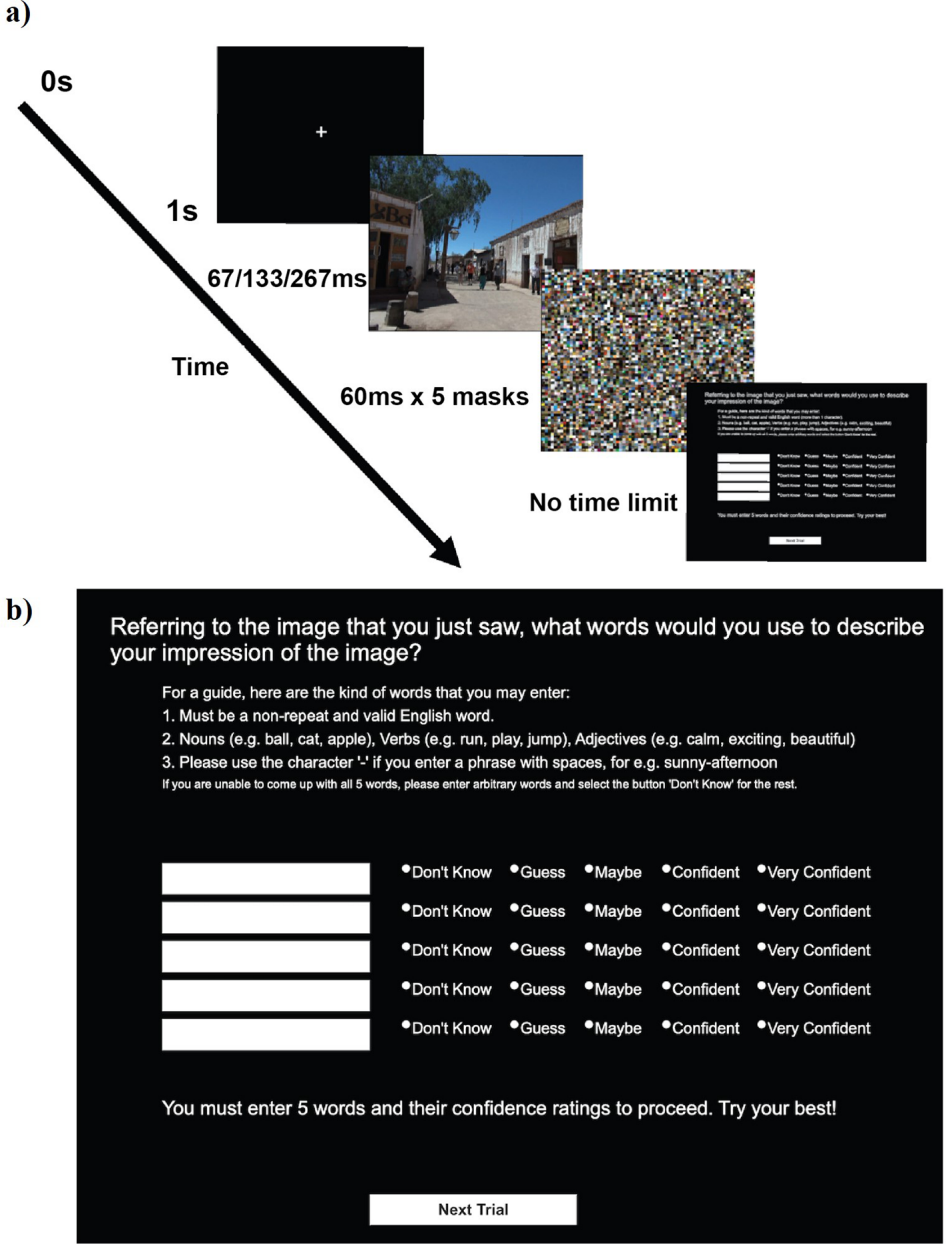
A single trial. a) Time course of a single trial. b) The response screen. The instruction said “For a guide, here are the kind of words that you may enter: 1, must be a non-repeat and valid English word; 2, nouns (e.g., ball, cat, apple), verbs (e.g., run, play, jump), adjectives (e.g., calm, exciting, beautiful); 3, please use the character ‘-’ if you enter a phrase with spaces, e.g., sunny-afternoon; if you are unable to come up with all 5 words, please enter arbitrary words and select the button ‘Don’t know’ for the rest”.

Figure 1 (a) explains the time course of one trial in our experimental paradigm (see also Methods). Upon brief exposure to an image, participants typed five words in the response box with confidence indicated in each word (See (b) in Figure 1). We tested 670 participants online, but analysed the first 30 participants within each of 20 groups. Thus, our results reflect those from 600 participants.

### Intersubjective agreement (IA)

To quantify the degree of “specificity” of a reported word on a given image, we compare the report frequency of the word between the target image and the rest of the images. We call the index “intersubjective agreement (IA)” because it describes how well the reported word agrees with what other participants reported on that image but not on the other images. We refer “Word IA” ^1^ to the IA associated with a particular word for a given image.

With our IA index, we do not need to assume any *a-priori* correct answers. A word response that has high Word IA with respect to an image indicates that the word is highly specific to the image, compared to the rest of the images.

### Calculation of Word IA

**Figure 2.**
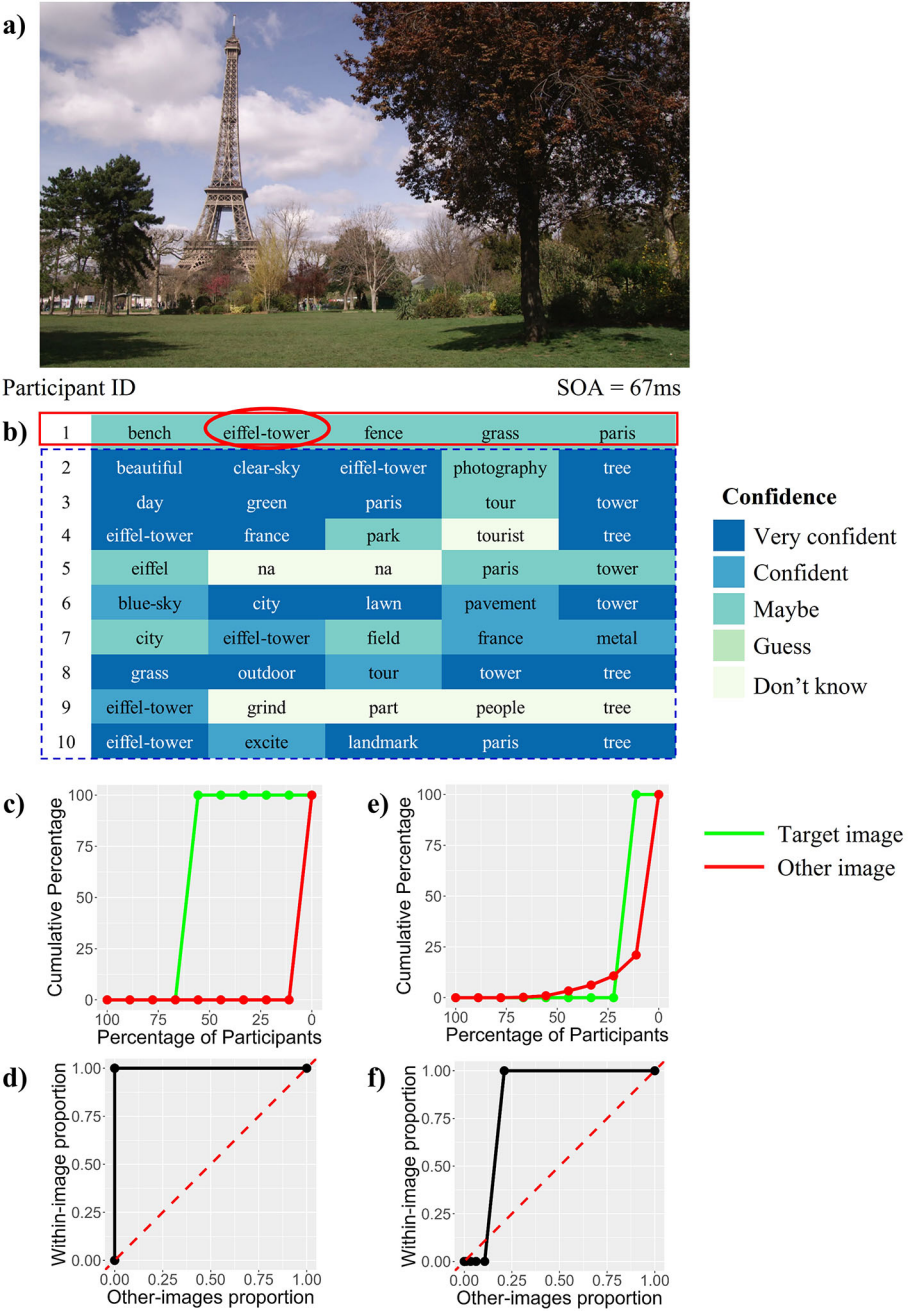
The example Calculation of Word IA. a) The image used in this example. b) The 50 words reported by 10 participants, color-coded by confidence ratings. SOA = 67 ms. c) Red line: The cumulative percentage (y-axis) of the other images (i.e., all other 419 images we tested) as a function of the percentage of participants who reported the target word (x-axis, note in the descending order from 100% from left to 0% to the right). Here the target word is “eiffel-tower”. This red curve serves as the baseline report frequency of the target word. The value on this curve doesn’t have to be 0 or 1, it jumps from 0 to 1 because no one reported “eiffel-tower” under the other images. Green line: it shows the same for the target image, which jumps from 0 to 1 at X = 56%, reflecting the fact that 5 out of 9 “other” participants who saw this image for 67 ms reported “eiffel-tower”. d) The Receiver Operating Characteristic (ROC) curve constructed based on two cumulative curves in c. To construct the ROC, we shifted the criterion. We plotted the cumulative percentage for a pair of the within-image (green) as y-coordinate and other-images (red) as x-coordinate across all criteria. e) & f) Another example for the target word “grass” after viewing the same image (a), whose Word IA = 0.84. They are constructed in the same way as (c) and (d). This example shows values on the red line are not binary.

Figure 2 explains the steps involved in calculating Word IA under a particular stimulus onset asynchrony (SOA). Word IA is defined on a particular image and a particular word. Let us explain the case using a target image in Figure 2 (a) and a word response “eiffel-tower” from participant 1 in Figure 2(b) (red solid box; For details, see Methods: Calculation of Word IA for each SOA). Figure 2(b) represents words reported by ten participants after viewing the target image for 67 ms.

Here, ‘eiffel-tower’ was reported by five out of nine “other” participants (= 56%).

Next, we estimate the baseline report frequency of the word “eiffel-tower” after seeing any one of the other images. Under SOA = 67 ms, no one reported the word “eiffel-tower” under the other 419 images.

We convert the count into percentages and calculate the cumulative percentages for within-image and other-image counts to obtain Figure 2(c) (green for the target image and red for the other images).

From this cumulative percentage, we construct the Receiver Operating Characteristic (ROC) curve (d) in Figure 2 and calculate the Area Under the ROC Curve (AUC). Supplementary Table 1 shows the cumulative percentage used to compute Figure 2(c) and (d). Figure 2(e) and (f) shows the cumulative curves and ROC curve of another response word “grass” after viewing Figure 2(a).

Finally, we repeat the steps above for each “eiffel-tower” reported by different participants under SOA = 67 ms and calculate the final Word IA value (SOA = 67 ms) of “eiffel-tower” under (a) in Figure 2 by averaging the AUC values.

Note that a word must be reported by at least one “other participant” (i.e., reported by two or more people in total) under the target image to have a valid Word IA value. The words that were reported by only one person are called “rarely reported words”. We will explain more later.

Similar to Word IA for each SOA, we can also calculate Word IA across SOA (see Methods: Calculation of Word IA across SOA for details). ^2^ In the following sections, we will first introduce the results from Word IA across SOA. This measures the specificity of response words robustly based on as many observations for each image. After that, we will compare Word IA between SOAs.

### Word IA are very high across images

**Figure 3.**
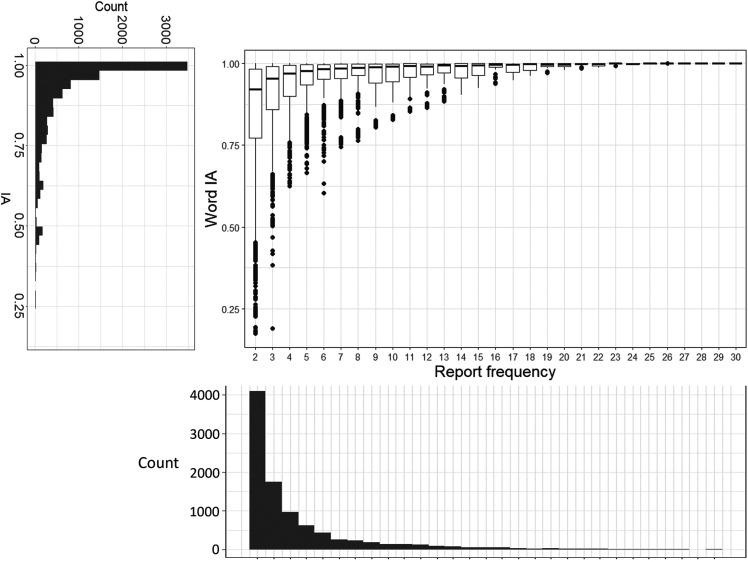
The distribution of Word IA and report frequency of each word. X axis is the report frequency of a word under a certain image (maximum = 30). Y axis is the Word IA values. The overall distribution of report frequency and Word IA is shown next to the axis.

As we mentioned above, Word IA ranges from 0 to 1. Most words that were reported had very high Word IA (close to 1) across SOAs. Over 9,277 words and 420 images, Word IA was distributed with the mean (± std) as 0.9 (± 0.14) and median (25%, 75%) as 0.96 (25%-tile 0.86 to 75%-tile 0.99) ( Figure 3). Thus, upon seeing a totally unexpectable image, people freely report five unique words that are rarely used to describe any other images in our set.

A traditional account of rapid scene experience predicts that people perceive a global and coarse description of the image upon brief seeing. If participants’ vocabulary is strongly confined to those global and coarse descriptions, Word IA should be low because those descriptions would be shared across images.

However, upon examination of each reported word in Figure 2 as well as Figure 3’s distribution, this is unlikely the case. First, Figure 2(b) contains the words that would be normally regarded as global and coarse gist descriptions (e.g., clear-sky) but also local and specific words (e.g., eiffel-tower, paris). While the former types of descriptors are expected to be reported for the rest of images, the latter are unlikely to be reported. Thus, Word IA should be lower for the former and higher for the latter. Very high Word IA in Figure 3 implies that participants’ vocabulary is not confined to global and coarse descriptions.

To understand how the nature of the target image affected Word IA, we calculated the mean of Word IA (across SOA) for all words reported on a particular image. We call it Image IA. Image IA estimates the consistency and selectivity of the reported words for a particular image.

**Figure 4.**
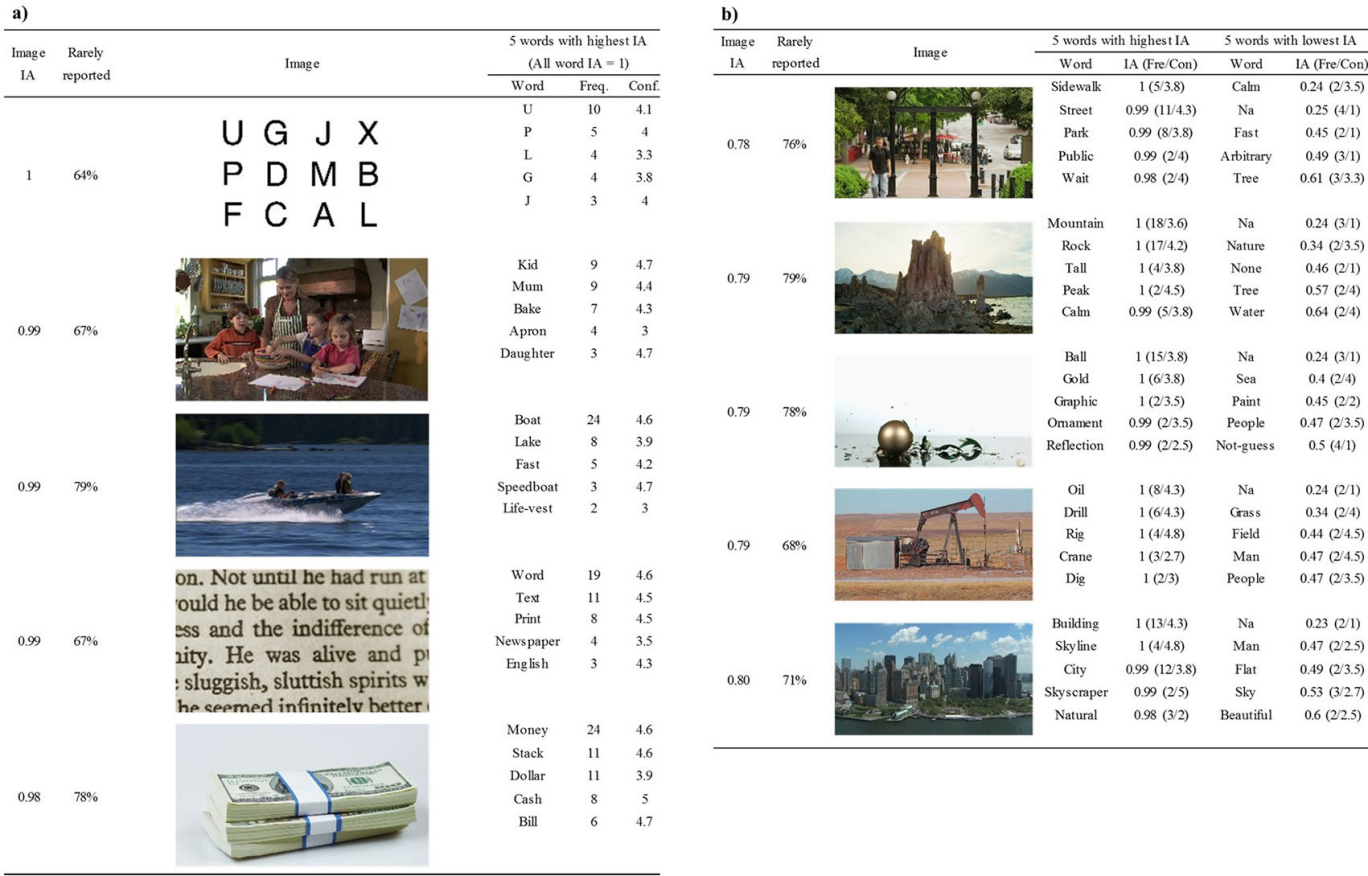
Images with highest (a) and lowest (b) Image IA. Five words with highest and lowest Word IA are also shown. The Word IA of all response words in (a) is 1. The figure shows that both images with highest and lowest Image IA have words whose Word IA is close to 1, accompanied with high confidence rating. The images in (b) have lower Image IA because they also have words with low Word IA. Fre. means the frequency of the word being reported (maximum = 30). Con. means the mean confidence ratings of the word across participants who reported it.

Figure 4(a) and (b) list the five images that had the highest (and lowest) Image IA (across SOAs) among 420 images, respectively.

The five images in Figure 4(a) had the mean Word IAs across all reported words to be 0.98 to 1. This means that a set of reported words for each of these images were extremely specific and almost never used to describe any of the rest of the images.

For example, the top image with Image IA = 1 was the letter array, typically used in the so-called Sperling paradigm in psychology ( Sperling, 1960). This type of image is often used to demonstrate the limit of what people can report upon seeing an image in a brief moment. Here, the reported words are almost always a single letter (e.g., U, P, L, etc) that was actually contained in the array. Note that these words were reported spontaneously without warning participants that they will be tested with these types of arrays. In fact, if anything, this image was included among 20 other natural scene images, thus participants would have had very low expectation in seeing anything like this (see Discussion: Elimination of expectation).

The rest of the top images with high Image IAs were all natural scenes. Among those words, although there are a few arguable ones, such as Text (11 out of 30 people reported) and Word (19), most words were local and detailed, including: Mum (9), Apron (4), Boat (24), Speedboat (3), and Life-vest (2), etc.

Figure 4(b) lists the five images that had the lowest Image IAs, ranging from 0.78 to 0.8. These scenes appear difficult to describe in words compared to the top images in Figure 4(a). Nonetheless, each image was reported with words whose Word IA was close to 1. This means that people agreed in describing these images with these highly specific words. Some of those words might be argued as global and coarse, such as Street (11), Tall (4), Natural (3) and City (12), whereas other words are more local and detailed, such as Ball (15), Crane (3), Dig (2), Drill (6), and Skyscraper (2). For these global and coarse words in Figure 4(b), we surmise that there are not many salient visual objects for detailed reports in the images. Thus, participants resorted to coarse descriptions, which are strongly shared by other people. As a result, the report frequency of these coarse descriptions are much higher than those for the rest of images, attaining high Word IA values.

Lower Image IA for Figure 4(b) is due to the result of many words that have lower Word IA, which were included to compute Image IA. There are some global and coarse words that can also be used to describe other images, such as Nature (2), Water (2), Sea (2), and Sky (3). There are also some meaningless words whose confidence rating is 1, such as “Na”, “Arbitrary”, and “None”, which are shared across images (i.e., low Word IA). The presence of these words indicates that some participants found it hard to describe the images using five distinct words. This is either due to the difficulty of the description of the image per se or due to the restriction of image viewing time, which we will investigate next.

Comparing the words whose Word IA is close to 1 in Figure 4(a) with those in Figure 4(b), we notice that the confidence ratings in Figure 4(a) tend to be higher than those in Figure 4(b). But the confidence ratings of the words with highest Word IA are much higher than those of the words with lowest Word IA within Figure 4(b), implying some correlation between confidence ratings and Word IA, which we will investigate next as well.

We also analyzed the proportion of rarely reported words (the second column from the left for Figure 4(a) and (b). They were not included in the calculation of Image IA because their Word IA was not defined. If a word was reported by only one person, it could be truly a unique word to describe that particular image, but it could also be a random response that was not elicited by the stimulus images. A higher proportion of rarely reported words could indicate that it is harder to perceive describable information from the image. To test that, we computed the proportion of rarely reported words for each image, but we found no correlation between the proportion and Image IA, r = -.06, df = 418, p = .195, 95%CI = [-0.16, 0.03]. Whether rarely reported words are not random responses can be addressed in the future studies by recruiting a larger number of participants.

### Word IA reflects mean confidence ratings for each word

As implied in Figure 4, high Word IA tends to be associated with high mean confidence ratings. If this correlation is reliable, Word IA can be taken as a proxy of conscious perception, reflecting metacognitive access to what participants see phenomenally ( Matthews *et al.*, 2018; Seth *et al.*, 2008).

**Figure 5.**
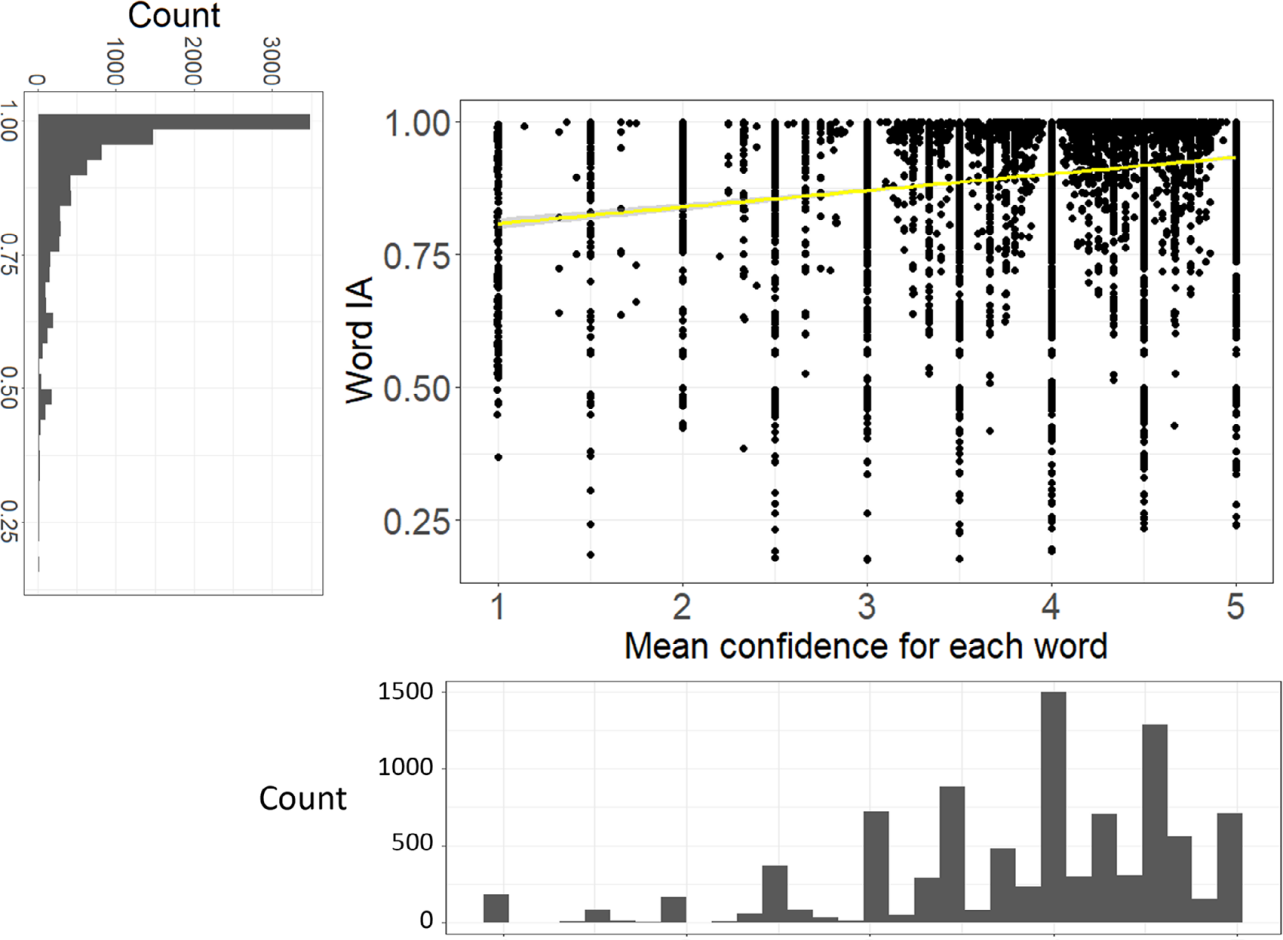
The higher Word IA (across SOA), the more confident people report with the words. X axis is the mean confidence (across participants who reported the word) for each word. Y axis is Word IA. 95% CI is also shown (gray shade).

Figure 5 shows the scatter plot of Word IA (across SOA) and mean confidence rating for each word, with the distributions of those variables. There was a significant correlation between Word IA and mean confidence rating for each word, r = 0.19, df = 9275, p < .001, 95%CI = [0.17, 0.21]. Thus, our Word IA measure captures an aspect of conscious perception, that is, the degree of confidence they have in reporting what they see.

### Even at the shortest SOA, Word IA correlates with confidence

It is plausible that the association between high Word IA and high confidence rating in Figures 4 and 5 are artefacts of our grouping of SOA. At longer SOAs (133 or 267 ms), it is likely that participants see details clearly and consciously with high confidence with high IA. At short SOA (67 ms), they might not see much and report random words with low confidence with low IA. Such results would inflate the correlation between Word IA and confidence ratings. To rule out this possibility, we examined the correlation at each SOA.

**Figure 6.**
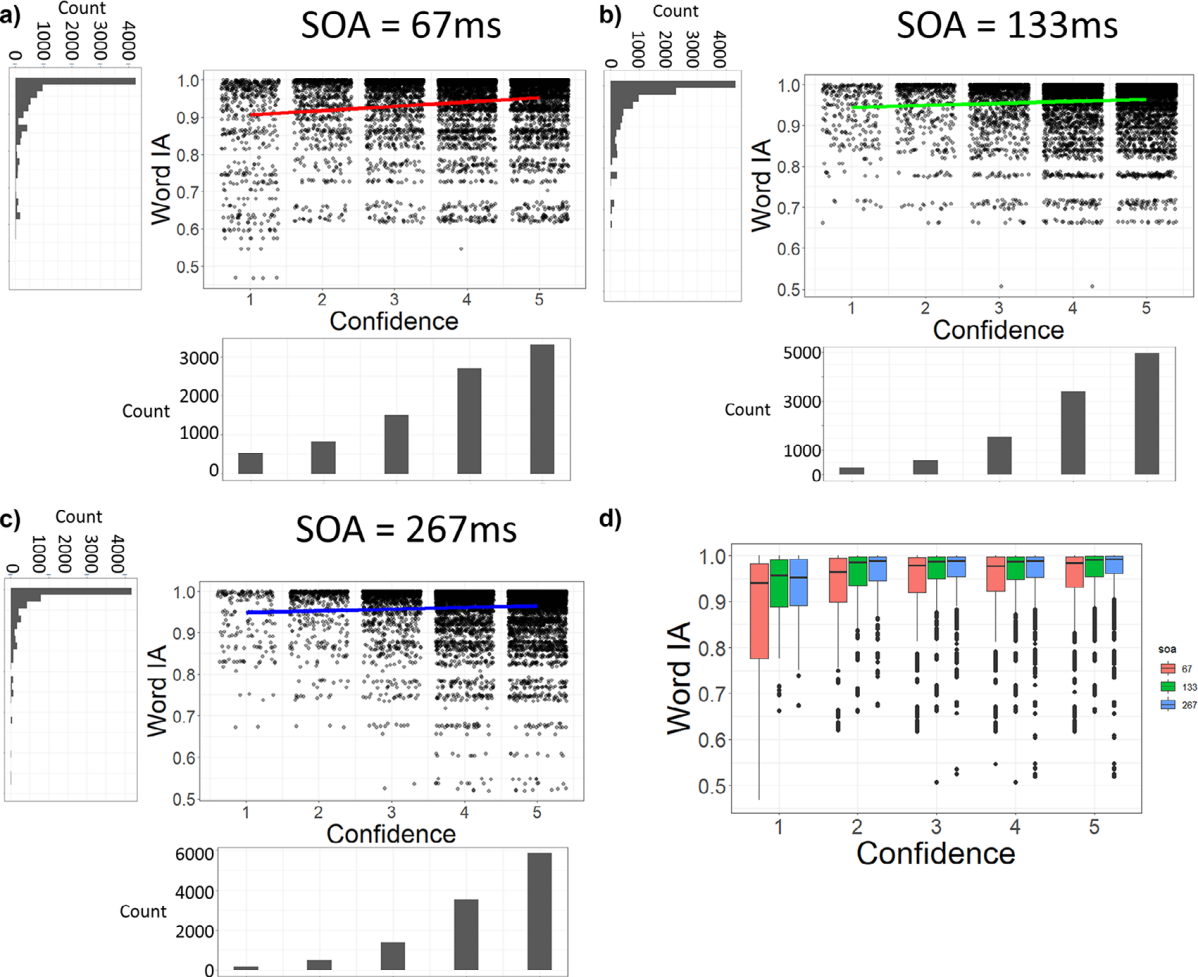
Confidence rating is correlated with Word IA at all three SOAs. a-c) The dot plots with jittering for confidence ratings. Note here, each dot represents a confidence rating from one participant for a given word. Word IA at SOA = 67 ms (a), 133 ms (b), and 267 ms (c). X axis is confidence rating. Y axis is Word IA. d) The boxplot showing Word IA for each SOA, grouped by confidence and SOA.

However, even under the shortest SOA (= 67 ms), there was a significant correlation between Word IA and confidence rating: r = 0.16, df = 8897, p < .001, 95%CI = [0.13, 0.18] ( Figure 6(a)). Figures 6(b) and (c) show the data for longer SOAs, which also showed significant correlations between confidence and Word IA: at SOA = 133 ms, r = 0.08, df = 10800, p < .001, 95%CI = [0.06, 0.10], and at SOA = 267 ms, r = 0.06, df = 11468, p < .001, 95%CI = [0.04, 0.08]. Therefore, confidence rating was weakly correlated with Word IA at all three SOAs.

To control the difference among participants and images, we analysed the data with four multilevel regression models with the random effects being intercepts for participants and images. Our first model (Model 1) is the null model which only included the random effects of intercepts, adjusted Intraclass Correlation Coefficient (ICC) = 0.120, AIC = 78094, BIC = -78061. Our second model (Model 2) added the fixed effect of confidence rating on top of Model 1, adjusted ICC = 0.119, AIC = -78513, BIC = -78471. It showed that Word IA for each SOA can be explained by confidence rating, p < .001. The third model (Model 3) added the fixed effect of SOA on top of Model 2, adjusted ICC = 0.120, AIC = -78825, BIC = -78775. It showed that Word IA for each SOA can be explained by both confidence rating (p < .001) and SOA (p < .001). The fourth model (Model 4) is the full model which added the fixed effect of interaction between confidence rating and SOA on top of Model 3, adjusted ICC = 0.120, AIC = -78924, BIC = -78866. It showed that Word IA for each SOA can be explained by confidence rating (p < .001), SOA (p < .001), and the interaction between them (p < .001). According to the AIC and BIC of these models, Model 1 is the worst model, whereas Model 2, 3, 4 are similar, with Model 4 having the lowest AIC and BIC. However, according to the likelihood ratio test ( Winter, 2013), there was a significant difference between Model 2 and 3, χ ^2^ = 314.23, df = 1, p < .001. There was also a significant difference between Model 3 and 4, χ ^2^ = 101.6, df = 1, p < .001. Therefore, Model 4 is the most preferred model, whose parameters are shown in Supplementary Table 2. The model shows that a higher SOA predicts higher Word IA.

The relationship between Word IA and SOA is visualised in Figure 6(d). Interpretation of results for confidence = 1 (about 5% of all responses) is somewhat complicated, because we explicitly asked participants to enter random words with confidence = 1 when they can’t give five words. Entries such as “none” and “NA” are often entered with confidence = 1. Nonetheless, the median Word IAs at confidence = 1 are still very high (higher than 0.9 for all SOAs), possibly reflecting fleeting impressions that participants felt, which were actually shared with other participants in specific and selective ways as captured by Word IA. When confidence is higher than 1, Word IAs were nearly saturated (median > 0.95) for all SOAs. When we exclude responses with confidence of 1, there is still a significant positive correlation between SOAs and Word IA (p < 0.001, found by multilevel regression).

### Beyond global and coarse reports revealed by Word IA – analysis of the highly similar image pairs and artificial images

Finally, we present somewhat unexpected, yet striking findings based on further analyses of two sets of images: highly similar image pairs and artificial image pairs.

**Figure 7.**
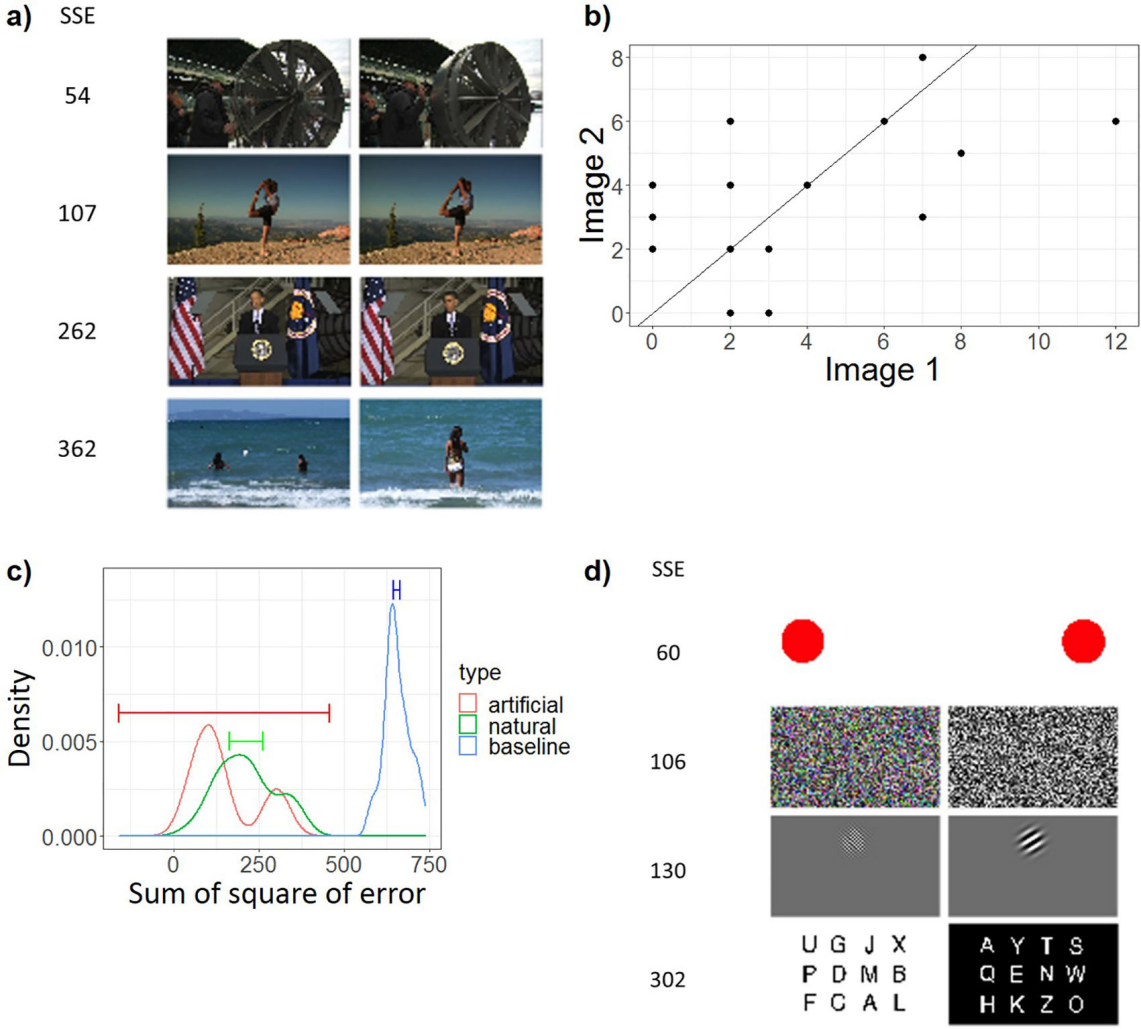
The Analysis of Highly Similar Images. a) Four example pairs of the highly similar natural images, including the pairs with lowest (54) and highest (362) Sum of Square of Error (SSE). For all highly similar natural images, see Supplementary Figure 2. b) An example plot of the frequency of all the unique response words from a pair of highly similar images. X axis is the frequency of response words from one image obtained from a set of N=30 participants. Y axis is the frequency of response words from the other similar image obtained from a separate set of N=30 participants. Each data point means a response word. The solid line is y = x, which means perfect consistency. c) The distributions of Sum of Square of Error (SSE) of pairs of two similar natural images (green) and artificial images (red). As baseline (blue), we computed SSE for randomly selected two images. X axis is SSE, which is the sum of squared distance between each data point and the y = x line in (b). Y axis is the probability density. 99%CI is shown by the line segment. d) SSE computed for four pairs of artificial images.

As explained in Methods, we filtered the identical images over the available image sets (screenshots from videos) with both automatic and manual procedures. However, unintentionally, we retained 22 pairs and two trios of highly similar but slightly different natural images; see a) in Figure 7 and Supplementary Figure 2. Rather than removing the data from these images, we analyzed them with our analysis method to test its validity.

For simplicity, we removed one image from each trio and obtained 24 pairs of natural images in total. To quantify the degree of the similarity of free reports between two similar images, we computed “sum of square of error (SSE)”. In short, SSE quantifies the similarity of the frequency of reports between two images presented to two separate populations of participants (see (b) in Figure 7). SSE is 0 when the reports are identical. It is expected to become large if it is computed between two different images.

The green line in (c) in Figure 7 demonstrates that two groups of participants gave a very similar set of words for these two similar images. This contrasts with the baseline (blue), where SSE is computed between randomly paired two images (for details, see Methods - Data analysis - shuffle the pairs of similar images). The fact that two distinct groups of N=30 participants responded highly similarly to the pairs of highly similar images lends support to our assumption for the IA measure: upon seeing similar images, a population of people give similar responses even when measured with an unconstrained free-report paradigm.

Secondly, we analysed the four pairs of artificial images (see (d) in Figure 7 and Supplementary Figure 3 for details), which have been used in previous psychophysical experiments. ^3^ Unlike natural images, we suspected that if these artificial images are presented to participants without any warning or expectation, they may not be able to see the contents very clearly (see Discussion). As we expected, participants did not really spontaneously report particular details of each simple image, which differentiates them (e.g., left vs. right, colored vs. black-and-white, high vs. low spatial frequency, left- vs. right-tilted). This is totally understandable given that these features only make sense when these pairs were presented in contrast and participants are invited to report their differences. Their spontaneous reports are more to do with the common attributes for these pairs, resulting in lower SSE.

One exception to this rule was the pair of the Sperling letter array, one of which was indeed featured in Figure 4(a), which achieved the highest Image IA among all the tested images. We expected that the dominant responses for these types of the images would be global and broad descriptions, such as “letters”, “alphabets”, but rarely the actual single letters, such as “U” or “A” ( Haun *et al.*, 2017). Contrary to our expectation, each letter was reported rather frequently as shown in Figure 4(a) and Supplementary Figure 3. This is quite remarkable, given that in our experiment, participants were never told about this type of stimuli (see Discussion: Elimination of expectation). As a result, the SSE between the paired Sperling images (= 302) was much larger than the other three artificial image pairs (60-130).

## Discussion

In the present study, we used a free-report paradigm ( Figure 1) to examine a widely-supported view on rapid scene experience. In a traditional view, people are expected to process and report coarse, but not detailed, information about a briefly presented scene. Rather than presuming what words should qualify as “correct” answers, we used the words that other participants reported about the same image as the expected report. Based on this notion, we proposed a novel measure, termed “intersubjective agreement” (IA) ( Figure 2). Word IA quantifies the specificity of a response word, by comparing the frequency of the response word under a given image with that under all other images. The frequency of the latter serves as the baseline report of the response word. With our IA measure, we demonstrated that participants report words that are maximally specific (IA = 1) across SOAs (as short as 67 ms) ( Figures 3, 4). Further, we demonstrated Word IA computed across SOA and at a given SOA showed correlation with confidence ratings, thus reflecting the degree of conscious access ( Figures 5, 6). The analysis of highly similar images ( Figure 7) assured the validity of our paradigm. Our results challenge the notion that reports on a briefly viewed scene are coarse and not detailed.

### Methodological novelties

**Free-report paradigm with our novel IA measure.** While the forced-choice tasks have revealed a great deal about various limits and scopes of the human visual system in rapid vision, they do not allow us to make inferences as to whether participants perceive more than what experimenters expected. Importantly, our paradigm is free from such a restriction imposed by us, the experimenters.

Our study is not the first application of the free-report paradigm to examine the nature of rapid visual perception. In a pioneering study, FeiFei *et al.* (2007) asked participants to write a short paragraph to describe the image of everyday scenes after briefly viewing it for 27 ms to 500 ms (masked). The responses were then analyzed by 5 scorers. Unfortunately, they introduced experimenters’ bias at this evaluation stage. Specifically, the experimenters asked the scorers to evaluate if the response paragraph contained objects that they, the experimenters, defined for the scorers. As such, the objects that were not included in the experimenters’ preset objects were not analysed.

Another issue of such assessor-based scoring is that the subjective experience of scorers is quite different from that of participants. In FeiFei *et al.* (2007), the raters saw the target image for a long duration moving their eyes to inspect various parts of the images. However, the participants in the main experiment saw the images briefly with minimal saccades. Thus, we cannot interpret what the participants actually experienced based on the scorers’ evaluations.

We minimised these sources of experimenters' biases and confounds. We analysed all that participants reported about their experience based on what other participants reported in the same viewing condition. Importantly, our novel strategy can scale like forced-choice paradigms without manual inspection. With more participants per image set, our estimate becomes more robust.

Although not directly comparable, some of the previous forced-choice paradigms studied aspects of rapid scene experience. For example, Biederman *et al.* (2006) and Larson *et al.* (2014) showed that participants’ responses were more accurate under a longer SOA. Similarly, in our study, Word IA for each SOA was positively associated with SOA (Supplementary Table 2). Importantly, this increase in IA was also accompanied with the increase in confidence and frequency of reports, consistent with previous studies as well (e.g., Fu *et al*., 2016).

To enhance these strengths, we employed an online experimental platform to recruit a large number of participants and to use a large set of stimuli ( Qianchen *et al.*, 2022). They are important prerequisites of our novel IA measure and our free-report paradigm. Note that in our preliminary experiment, we did not see any qualitative difference in the results between in-lab participants (N=10) and online participants (see Supplementary Figure 4).

**Elimination of expectation.** Another important extension is that our paradigm does not allow participants to expect what category of images will be shown. In previous studies, participants were told that the images would be one of a few categories (e.g., animal vs. non-animal; Fabre-Thorpe *et al.*, 2001). High accuracy in rapid scene processing (e.g., Evans & Treisman, 2005; Fabre-Thorpe *et al.*, 2001) may depend on expectation ( McLean *et al.*, 2021; Sun *et al.*, 2017). In our paradigm participants could not expect the images, yet they still reported impressive details of the briefly presented images.

**Confidence rating correlates with Word IA.** Surprisingly, the detailed information accompanied with high confidence across images ( Figures 4, 5 and 6).

The results with Sperling arrays (see Figure 4) were rather notable. Without any expectations, participants reported specific letters, which goes against an idea that conscious perception strongly depends on expectation ( Mack *et al.*, 2017; Pinto *et al.*, 2015). Note these specific letters reported with high confidence were the ones that were included in the array.

Speaking of confidence rating, we are not aware of other studies that asked participants to provide confidence ratings together with words in a free-report paradigm. Our study is perhaps the first that combined a free-report and confidence rating. Probably because of the saturation of Word IA, its correlation with confidence was weak, but the words with high confidence were still more specific and shared between participants ( Figures 4, 5, and 6). If we consider what participants reported in this study as a “gist” of a scene, we can interpret it to mean that gists are something that we can metacognitively monitor and consciously access. In light of past studies that showed gists can be grasped without attentional amplification ( Li *et al.*, 2002; Mack & Rock, 1998), this further supports the notion that conscious perception does not always require attentional amplification ( Koch & Tsuchiya, 2007; Tsuchiya & Koch, 2016). Our study extends this notion with freely-reported image contents without expectation.

Importantly, we observed highly specific words (IA = 1) even in SOA = 67 ms condition (77% of images). Many specific words came with the highest confidence of 5. Non-specific words (IA < 0.6) were observed much less frequently across SOAs (30% for SOA = 67 ms, 19% for SOA = 133 ms, 17% for SOA = 267 ms). We predict this pattern of results would not be observed if the image was strongly masked to make the image invisible ( Kaunitz *et al.*, 2011). Taken together, our findings require the revision of rapid scene experience from traditional “coarse and nonspecific” to “detailed and specific even without expectation”.

### Repetitions of images

What is the potential source of the discrepancy between ours and traditional findings? Given that we obtained similarly high IA for both artificial (letters) and natural stimuli, the source of discrepancy is unlikely to be solely due to the type of the stimuli.

Instead, we point out another important feature of our task. In our task, we never repeated the same image for a given participant. We suspect this is one source of the discordance as previous studies tended to use the same stimuli across trials (e.g., Kimchi, 1992; Navon, 1977). As Endress and Potter (2014) suggested, repeated presentation causes prospective interference, possibly biasing our attention and expectation to the features that distinguish trials (see also Kaunitz *et al.*, 2016; Qianchen *et al.*, 2022). It is worth revisiting previous studies but using stimuli that are never repeated across trials.

### Presentation duration

What is the minimal duration that is required for such rapid recognition? When we designed this study, we were not confident if the online study can achieve reliable presentation of short duration stimuli. Thus, we opted for going rather conservative durations of 67 ms as minimal. Further, we did not expect that participants could make detailed word reports that correlate with confidence. Therefore, we did not push the limit. Future studies can test much shorter duration.

We performed some preliminary experiments with shorter SOAs online and confirmed its feasibility. To conduct such a study in the future, however, we recommend to confirm the results with in-lab participants to some extent. We also suggest that shorter SOA trials should be intermixed with longer SOA trials, so that participants can set an appropriate threshold for reports ( Lin & Murray, 2014). Such experiments will address an important question on the temporal limit of intersubjectively shared experience upon viewing rapid visual scenes. 

### Future directions

Our novel paradigm with IA measure is highly adaptable for different research purposes. For example, any future researchers can use our current data as the normative data (our data is fully and freely available online for both the image sets [ https://osf.io/q2cr8/] and the response words [ https://osf.io/7spxd/]). Using them as the baseline, it would be easy to perform a study to test if some population of patients with known mental disorders, such as schizophrenia, would see and report the same words as the healthy population. Future studies could also utilize different stimulus sets to address different questions. For example, if we change the nature of the stimulus set into emotional movies (e.g., Cowen & Keltner, 2017), we can characterize commonality and differences of the nature of emotional experiences using free report paradigms without imposing any theoretical assumptions introduced by us, the experimenters. Also, the emotional intensity within each movie can be calculated using normative ratings of lexico-semantic factors (e.g., valence and arousal; Mohammad, 2018).

Finally, our paper has some implications to the theoretical debate on whether conscious experience is restricted by cognitive access or overflows such restriction ( Block, 2007; Bronfman *et al.*, 2019). Previous debate on the overflow issue was almost always focused on the artificial stimulus situation introduced by Sperling (1960). Our paper contributes to this debate in two fresh ways. First, since the Sperling’s initial introduction of the paradigm, it has been almost always “assumed” that what participants see in the “whole-report” condition is just a gist, such as “seeing everything”, “alphabets”, and “letters” ( Haun *et al.*, 2017). Our empirical free-report challenges this notion. Even without any warning or expectation, participants report specific letters rather than generic gists (e.g., Figure 4a). Second, our paradigm opens up a possibility to examine the overflow issue with naturalistic stimuli. For that, we would need to develop equivalent experimental contrasts between the whole- vs. partial reports as in Sperling (1960), which is not impossible.

## Conclusion

Contrary to a widely supported view that participants are more likely to perceive coarse information from a briefly presented natural scene image, we found that participants reported highly detailed descriptive words with high confidence. Our findings show that even without expectation, a brief glance of scene images is enough to provide us with a vivid conscious experience which can be metacognitively monitored. Our novel paradigm and measures can lead to an exciting avenue for future research that finds intersection on sensory psychology, big data, cognitive-linguistic and consciousness research.

## Methods

### Participants

We recruited 801 online participants from Amazon’s Mechanical Turk (MTurk) who had a Human Intelligence Task (HIT) approval rating of more than 97% and more than 5,000 approved HITs. Among them, 131 participants did not complete the task and were excluded, leaving 670 valid participants. All the analysis presented in this paper is based on the first 30 participants for each of 20 groups (see below for details), that is 600 participants. MTurk has demonstrated its reliability and validity ( Sheehan, 2017) by replicating lab effects in many research areas ( Amir *et al.*, 2012; Crump *et al.*, 2013; Klein *et al.*, 2014; Paolacci *et al.*, 2010). Participants read the online explanatory statement and gave informed consent by pressing a key before they began the experiment. Each participant received US$3 as compensation for their time (30 minutes). Upon completion of the experiment, participants answered a few demographic questionnaires. Within the questionnaires, participants were allowed to answer ‘NA’ if they wished. We used TurkPrime ( Litman *et al.*, 2017) to manage the recruitment and payment of participants from MTurk. Supplementary Figure 5 shows the demographics (sex, age, nationality, first and second language, number of years speaking English) of the participants. Ethics approval was obtained from the Monash University Human Research Ethics Committee (approved project ID 17674).

### Apparatus

To create the online experiment, we used InquisitLab and InquisitWeb (version 5; Software, 2018). Inquisit’s software provides highly accurate stimulus presentation time (within milliseconds; De Clercq *et al.*, 2003). To minimise the effect of a slow network, we asked participants to download the software package of InquisitPlayer, which then downloaded all experimental files to their computer before the experiment. During the experiment, InquisitWeb blocked the participant from interacting with other software programmes on the same computer, reducing the likelihood of poor data quality due to distractions. The functions of above software can be equivalently performed by PsychoPy and Pavlovia.

### Stimuli

One of the authors (SN) captured 9120 still-frame naturalistic images from a series of online videos at Videoblocks. Each coloured image was in JPEG format with a resolution of 1920 × 1080 pixels. As a video contained multiple still-frame images captured within a one-second interval, some pictures were highly similar. After filtering the identical images, we obtained 570 images.

Among these images, we randomly selected 415 images for our experiment (shown in Supplementary Figures 6a - 6f). Among them, three images were used as practice images. In addition, we included eight images (four pairs) of artificially generated images to represent typical stimuli used in psychophysics experiments (i.e., random noise, Gabor patch, Sperling letter array ( Sperling, 1960), a red circle; see (d) in Figure 7 and Supplementary Figure 3). As a result, we had 420 experimental images and three practice images. We randomly divided the experimental images into 20 blocks each with 21 images. Each participant saw the three practice images and a block of 21 images.

### Procedure

Upon downloading the InquisitPlayer, the experiment began with a consent form. After giving the consent, participants received an instruction on how to perform the task. They were told that in each trial, they would briefly see an image. Their task was to enter five valid English words without any repeats to describe their impression of the image. They were allowed to use nouns, verbs, and adjectives. They were reminded of this instruction while they typed the words in each trial (see (b) in Figure 1). Upon these instructions, participants practiced the task for three trials (with the same three images across participants). The stimulus onset asynchronies (SOAs) for these trials were randomly assigned from 67, 133, or 267 ms, one trial each. These images were not analysed.

Each participant was tested in one block that contained 21 trials. Figure 1 shows the time course of a single trial, which started with 1 second of fixation, followed by a target image. The target image was shown for a variable duration until the five successive masking images, each presented for 60 ms. SOA between the target and the first mask was either 67 ms or 133 ms or 267 ms. Each mask was an image with 1920 × 1080 pixels, which we constructed by filling it with 16 × 16 pixel image patches randomly taken from the 420 experimental images.

After the mask, participants were presented with the response screen (see (b) in Figure 1). Participants gave response words as instructed and rated how confident they were in seeing each word in the image (1: Don’t Know, 2: Guess, 3: Maybe, 4: Confident, 5: Very Confident). The response screen restricted participants to enter only alphabets and numerics. They were not allowed to use the empty space and asked to use a hyphen (‘-’) instead. If participants could not come up with 5 words, they were instructed to enter an arbitrary word with “Don’t know” as the confidence rating. ^4^

For each participant, we tested seven trials for each SOA (66 ms, 133 ms, and 267 ms), whose order was randomised across participants. Across 21 trials, participants never saw the same image more than once. For a given block of 21 images, we aimed to test a cohort of 30 participants so that each of the 21 images was presented to at least 10 participants at each SOA. Sometimes, MTurk recruited more than 30 participants. When this occurred, we took the first 30 participants’ data for the subsequent analyses. For this reason, 70 participants were excluded so we had 600 participants in our data analysis.

### Data cleaning

We performed minimal curation on the reported word as we did not want to inject our own bias into the data set. We provided all raw data so that researchers can examine the effects of any curation on our result. Our minimal curation procedure included the following.

If a participant entered the same word for a single image more than once, then we took only the first one with its confidence rating, and removed the second response (or later) from the analysis.

For each word, we applied three pre-processing steps. First, we converted the words to lowercase using the R package tm version 0.7-8). Second, we corrected misspelled words semi-automatically using the R library hunspell version 3.0) package (if the spell checker returned with one suggestion, we adopted it if relevant. Otherwise, we manually inspected the alternate suggestions and picked the most appropriate one). Finally, we performed word-lemmatisation using the R-package textstem version 0.1.4). Word-lemmatisation grouped similar words (e.g. plurals, verb tense), such as “child” and “children”, “walked” and “walk”, into a single base-form word so that the subsequent analysis would consider them as the same word.

After all these steps, we obtained 63,000 word responses (i.e., 420 images * 3 SOAs * 10 participants * 5 words) for the subsequent analysis. After combining the same words under the same images and the same SOA, we had 41,170 unique words. These word responses along with associated confidence ratings are available as a CSV file at https://osf.io/7spxd/.

### Data analysis

**Calculation of Word IA for each SOA.** For each target image at a given SOA, there were 10 unique participants who saw it. For each of 50 reported words, we went through the following processes. First, we counted the number of participants out of the rest of nine participants who reported a target word. Second, for each of all other 419 images, at the same SOA condition, we counted the number of nine participants who reported the target word. We excluded one participant that has the same participant order for the other images to equate the number of the participants for this counting to be 9 per SOA. Third, based on the number of these participants, we computed % of report and % of cumulative report for both the target image and all other images (Supplementary Table 1). From these, we applied the logic of Signal Detection Theory ( Green & Swets, 1966; Macmillan & Creelman, 2004). We used the cumulative frequency of reports to construct the receiver operating characteristic (ROC; see (c) and (b) in Figure 2. Fourth, we calculated the area under the ROC curve (AUC). If there were more than one participant who reported the same target word, we went through the same procedure and computed the mean AUC across them as Word IA at that SOA.

As we explained in the Results section, if the target word was reported only by one participant among 10 participants, it is called “rarely reported word” and its Word IA is not defined. Among the 41,170 unique (word, image, SOA) groups, 30,954 of them were only reported by one person and therefore didn’t have a defined Word IA, which left us 10,216 response words with a valid Word IA.

**Calculation of Word IA across SOAs.** We also computed Word IA by pooling the responses across SOAs. We did this to reduce the possibility of rarely reported words and to estimate the baseline rate of the reported words more robustly.

We performed the same analysis as Word IA for each SOA with the following exceptions. For each target image across SOAs, we had 30 unique participants who saw it, resulting in 150 reported words. For Word IA across SOAs, we counted the number of participants out of the rest of 27 participants who reported a target word. We removed one participant from each SOA group so that across SOAs analysis is not biased. Similarly, we did so for 27 participants to estimate the baseline with the other images. Other aspects of the calculation were the same as Word IA for each SOA.

Among the 30,911 unique (word, image) groups, 21,634 of them did not have a defined Word IA, which left us 9,277 response words that had a defined Word IA.

**Exclusion of similar images in Word IA calculation.** Although we tried to filter highly similar images, we noticed there were still some highly similar (but slightly different) image pairs in our image set (Supplementary Figure 2). While none of these pairs was presented to the same participant, word responses generated from these images could affect Word IA. For a given reported target word, the Word IA for one of the similar paired images will be generally lower because the target word is likely to be reported under the other paired image. To reduce the effect of this problem, when calculating the Word IA of responses to one of these images, we excluded the paired image from the baseline.

**Multilevel regression models.** The models were performed using the “lme4” package in R. The parameters were estimated using the maximum likelihood (ML) method.

**Shuffle the pairs of similar images.** To compare the consistency between pairs of highly similar images with random image pairs (in Results section: Beyond global and coarse reports revealed by Word IA), we constructed a “null distribution” as the baseline using a bootstrap method. We randomly selected one image from each of the 24 pairs and paired it with another image that was randomly selected from the 48 natural images (i.e., the 24 pairs of highly similar images) to obtain 24 random pairs of images. We then calculated the SSE (see Results section) of each pair and the mean of the 24 pairs. We repeated this process 100 times to obtain the “null distribution” of SSE.

## Data availability

### Underlying data

OSF: Human Annotations with Confidence Ratings on Natural Images. https://doi.org/10.17605/OSF.IO/7SPXD ( Chuyin, *et al.*, 2021)

This project contains the following underlying data

gist_batch_v1_all_mt_raw.csv (the raw data)

gist_batch_v1_all_mt_summary.csv (all the participants who signed up the study on MTurk)

Data are available under the terms of the Creative Commons Attribution 4.0 International license (CC-BY 4.0).

### Extended data

OSF: Natural Scene Images for the IA paper. https://doi.org/10.17605/OSF.IO/Q2CR8 ( Nishimoto *et al.*, 2021).

This project contains the following extended data

587 natural scene images in JPEG.

8 artificial stimulus images in JPEG.

Data are available under the terms of the Creative Commons Attribution 4.0 International license (CC-BY 4.0).

OSF: Supplementary materials for the IA paper. https://doi.org/10.17605/OSF.IO/U6QPM ( Chuyin, *et al*., 2021).

This project contains the following extended data

Supplementary Tables and Figures.pdf (Supplementary Tables 1-2 and Supplementary Figures 1-7)

Supplementary tables.xlsx (Supplementary Tables in Excel)

Motivation of IA.pdf

Data are available under the terms of the Creative Commons Attribution 4.0 International license (CC-BY 4.0).

Experiment code available from: https://doi.org/10.5281/zenodo.5794712 ( Koh & mluu0010, 2021)

Processed data and analysis code available from: https://doi.org/10.5281/zenodo.5796303 ( Chuyin & Koh, 2021)

Data are available under the terms of the Apache license 2.0.

## Footnotes

^1^
To be precise, we should perhaps use “Word-Image IA” instead of “Word IA” as it links to an image-word pair. Just for the simplicity and readability, we use “Word IA”.

^2^
We described our motivations and rationale behind our definition of IA over other simpler methods, such as ratio of reported words for the target vs all images in Supplementary Material (see https://osf.io/nvfhs).

^3^
Because we had 412 natural scene images and only 8 artificial images (4 pairs), reported results about Word IA reflect mostly for natural images. We checked if any of the results changed significantly after removing artificial images, but they did not.

^4^
In a pilot task, we asked participants to report up to 10 words per image. This task turned out to be difficult or tedious for many participants. Most participants seemed to be comfortable in reporting roughly up to 5 words per image. As we wanted to obtain as many words per image as possible, we settled on the 5 words.
