# Supplementary material for: What can we experience and report on a rapidly presented image? Intersubjective measures of specificity of freely reported contents of consciousness
Source: F1000Res. 2024 Aug 23;11:69. Originally published 2022 Jan 20. [Version 3] doi: 10.12688/f1000research.75364.3 (PMC9493396; doi:10.12688/f1000research.75364.3)
Supplement: Peer review history

# Reviewer report: Reviewer response for version 2

**DOI:** 10.5256/f1000research.137770.r148424

**Published:** 2022-09-21

**Authors:** Qiufang Fu

**Affiliations:** 1 State Key Laboratory of Brain and Cognitive Science, Institute of Psychology, Chinese Academy of Sciences, Beijing, China

The authors have successfully addressed all my concerns. I would like to thank the authors for their thorough responses.

Is the work clearly and accurately presented and does it cite the current literature?

Yes

If applicable, is the statistical analysis and its interpretation appropriate?

Not applicable

Are all the source data underlying the results available to ensure full reproducibility?

Yes

Is the study design appropriate and is the work technically sound?

Yes

Are the conclusions drawn adequately supported by the results?

Yes

Are sufficient details of methods and analysis provided to allow replication by others?

Yes

Reviewer Expertise:

Implicit learning, subliminal perception,

I confirm that I have read this submission and believe that I have an appropriate level of expertise to confirm that it is of an acceptable scientific standard.

# Reviewer report: Reviewer response for version 1

**DOI:** 10.5256/f1000research.79226.r144465

**Published:** 2022-08-08

**Authors:** Qiufang Fu

**Affiliations:** 1 State Key Laboratory of Brain and Cognitive Science, Institute of Psychology, Chinese Academy of Sciences, Beijing, China

This study combined a free-report and confidence rating to investigate how detailed information people can report after briefly seeing an image. Each image was presentation for 67, 133, or 267 ms, and followed by a dynamic masks for 300 ms. Six hundred and seventy participants attended this experiment online. A novel IA (Intersubjective Agreement) was developed to quantify how specifically the response words were used to describe the target image. The results showed that participants reported highly specific and detailed aspects of the briefly shown image, and IA is positively correlated with confidence.

 I found that the novel paradigm is very interesting and the new findings are intriguing. However, as the method is novel, the authors might need to elaborate it more on the novel index and how the procedure can measure what they aimed to measure.

 My concerns are mainly as follows:

1. For each image, participants were asked to enter five words and reported their confidence for each word. Why were participants asked to report five words rather than one or two or three or four words? Is it possible that this manipulation led participants to give more specific information about each image as the scene gist was unique while there were various kinds of specific information? I am wondering whether there were a sequence effect of IA on the five words. For example, is it possible that the IA for the first word were less specific than that for the other words?
2. The authors used a wide array of natural and artificial images but did not report IAs separately for natural images and artificial images. However, the authors mentioned “the current study aims to introduce a novel methodology to investigate how detailed information participants can report after briefly seeing a natural scene image” in the abstract. Thus, it would be better to analyze IAs for natural and artificial images separately.
3. Three SOAs were adopted in the current study. Previous studies indicated the accuracy increased with longer SOA in the forced-choice paradigms. I am wondering how the IA changed with SOA in the current study?
4. Given that when MTurk recruited more than 30 participants for a given block of 21 trials, the authors took the first 30 participants’ data for the subsequent analyses. There were 20 blocks, and thus the data from 600 rather than 670 participants were included. Is it right? Would you please make this more clear.

Is the work clearly and accurately presented and does it cite the current literature?

Yes

If applicable, is the statistical analysis and its interpretation appropriate?

Not applicable

Are all the source data underlying the results available to ensure full reproducibility?

Yes

Is the study design appropriate and is the work technically sound?

Yes

Are the conclusions drawn adequately supported by the results?

Yes

Are sufficient details of methods and analysis provided to allow replication by others?

Yes

Reviewer Expertise:

Implicit learning, subliminal perception,

I confirm that I have read this submission and believe that I have an appropriate level of expertise to confirm that it is of an acceptable scientific standard, however I have significant reservations, as outlined above.

# Response

**Published:** 2022-08-21

**Authors:** Zhang Chuyin

**Affiliations:** Monash University, Australia

Thank you for reviewing our paper and providing valuable comments! We will respond to your concerns one by one.

 1, *For each image, participants were asked to enter five words and reported their confidence for each word. Why were participants asked to report five words rather than one or two or three or four words?*

**Reply: **We agree that the number of response words is a key manipulation in our paradigm. To be honest, it is not easy to answer this “why” question. We imagine that the results might have been different if we asked participants to report 1, 2, 3, or 4 words. As a novel task, we had to start from somewhere. 

 In the pilot version of the experiment, we asked participants to report up to 10 words per image. This task turned out to be difficult or tedious for many participants. Meanwhile, most participants seemed comfortable in reporting roughly up to 5 words per image (including an option of reporting random words with confidence rating of “Don’t know”). As we wanted to obtain as many words per image as possible, we settled on the five words. This information has been added as a footnote as follows: 

- Footnote: “In a pilot task, we asked participants to report up to 10 words per image. This task turned out to be difficult or tedious for many participants. Most participants seemed to be comfortable in reporting roughly up to 5 words per image. As we wanted to obtain as many words per image as possible, we settled on the 5 words.” 

We believe that most aspects of the results would be replicated even if we reduce the number of words into 4, 3, 2, or 1. But, which aspects of the results stay the same or differ will be an empirical question. Having said that, repeating all experiments in this paper with the reduced report number of words will consume a lot of time and resources. Thus unless we have a specific question to address, we hope not to include this in this paper. We hope the reviewer would agree with this.  

 2, *Is it possible that this manipulation led participants to give more specific information about each image as the scene gist was unique while there were various kinds of specific information? I am wondering whether there were a sequence effect of IA on the five words. For example, is it possible that the IA for the first word were less specific than that for the other words?*

**Reply: **Based on what we expect from the literature, we agree with the reviewer that participants may first report the scene gist, then more specific information. To test this, we directly analysed the sequence effect on Word IA as you suggested (see Figure R1a). Although Word IA are almost saturated to the maximum value of 1, we can still see Word IA tends to be slightly higher for earlier-reported words at all three SOAs with SOA = 67ms having strongest effect of the sequence (This is confirmed statistically: a negative correlation between report order and Word IA, r = -0.056, p < .001. The interaction between SOAs and report sequence is significant, p < .001). The report sequence didn’t influence confidence much even when we looked at separately for each SOA (Figure R1b). 

 Thus, if we assume that Word IA captures something about “specific information” (we believe it is, as it is only high when the word is reported to the target image, but not to the other images), the report sequence does not matter much (even at the 5th location with SOA = 67ms, median Word IA is > 0.96). Perhaps, to see the report sequence effects clearly, we may need to use threshold level natural scenes (e.g., with SOA = 17 or 33 ms) in the future study.

**See Figure R1 linked here**

**Figure R1.** Report sequence effect analysis. a) The boxplot showing Word IA, grouped by report sequence and SOA. b) Confidence, grouped by report sequence and SOA. 

 Because this finding is counter-intuitive and possibly going opposite of what has been established in the field using artificial stimuli, we checked if this result is true only for natural stimuli. Please be mindful that given we included only 8 artificial stimuli, the following analysis is limited. 

 In particular, we checked the two Sperling letter arrays (1960). With this letter array stimuli, many researchers would agree that individual letters, such as {U, P, L, G, J} in Figure 4a, are specific responses, whereas descriptions about more generic aspects, such as alphabet, letter, are more like scene gist. Thus, focusing on these images makes it easier to check the report-sequence vs specificity hypothesis. For both types of reports, we calculated the percentage of responses at each report sequence (see Figure R2). 

**See Figure R2 linked here**

**Figure R2. **The percentage of responses given at each report sequence for generic and specific responses. 

 Under our experimental condition, it appears that participants tend to report specific letters first, then generic impressions later. It is interesting to note that this result was obtained despite the fact that we included the Sperling array in the midst of natural scenes, which would not let participants to expect specific letters. Having said that, due to the limited amount of the data, a chi-square test found no significant association between report sequence and the type of response, *X ^2^* (4) = 6.03, p = .1966 (N = 30 participants for each of two images). Thus, this observation is quite preliminary. 

 In summary, we infer that there is a very weak sequence effect that participants tend to report specific words first. This won’t influence our main finding that participants are able to report very specific information at a brief glance of the images.

 3, *The authors used a wide array of natural and artificial images but did not report IAs separately for natural images and artificial images. However, the authors mentioned “the current study aims to introduce a novel methodology to investigate how detailed information participants can report after briefly seeing a natural scene image” in the abstract. Thus, it would be better to analyze IAs for natural and artificial images separately.*

**Reply: **We agree that natural images and artificial images might show different results. However, because we had 412 natural scene images and only 8 artificial images (4 pairs), reported results reflect mostly for natural images. However, the overall trends of the results are quite similar as we summarised in Figure 7. In Figure 4a, we also analysed the Sperling’s letter arrays in particular, because they have been widely studied by previous studies. Our Figure 4a shows that the letter array has the highest Image IA (mean Word IA under an image), and almost all the responses were very specific. Having said that, we checked if the results changed after removing artificial images. We confirmed that they did not change. We added this in results as follows: 

- Footnote: "Because we had 412 natural scene images and only 8 artificial images (4 pairs), reported results about Word IA reflect mostly for natural images. We checked if any of the results changed significantly after removing artificial images, but they did not."

4, *Three SOAs were adopted in the current study. Previous studies indicated the accuracy increased with longer SOA in the forced-choice paradigms. I am wondering how the IA changed with SOA in the current study?*

**Reply: **We note that in Fu et al.’s (2016) categorization task, participants’ accuracy increased from ~70% (SOA = 40ms) to ~90% (SOA = 213ms). Thus we checked to see if there is a significant relationship between SOA and Word IA, as a proxy of “accuracy” in our study (Note that Word IA should not be taken as “accuracy” in the usual sense, where experimenters define what the answer should be. Rather IA measures the agreement or similarity of experience among participants, which may or may not match with what experimenters’ expected answers). 

 We added a Figure 6d into the paper (see Figure R3) to show this relationship more clearly. Interpretation of results for confidence = 1 (~5% of all responses) is somewhat complicated, because we explicitly asked participants to enter random words with confidence = 1 when they can’t give five words. In fact, entries such as “none” and “NA'' are often entered with confidence = 1. For SOA = 267ms, the median Word IA is around 0.5 when confidence = 1. This is consistent with an idea that participants know that their report does not reflect the image. For SOA = 67ms and 133ms, however, the median Word IA are higher than 0.5 (0.65 and 0.82, respectively), possibly reflecting fleeting impressions that participants had, which were actually shared with other participants in specific and selective ways as captured by Word IA. When confidence is higher than 1, Word IAs were nearly saturated (median > 0.95) for all SOAs. (There is a significant positive correlation between SOAs and Word IA  (p < 0.001, multilevel regression), when we exclude confidence = 1).

 Perhaps, to see the SOA effect clearly, we may need to use threshold level natural scenes (e.g., with SOA=17 or 33 ms) in the future study.

**See Figure R3 linked here**

**Figure R3 (Figure 6d).** The boxplot showing Word IA, grouped by confidence and SOA.

 5, *Given that when MTurk recruited more than 30 participants for a given block of 21 trials, the authors took the first 30 participants’ data for the subsequent analyses. There were 20 blocks, and thus the data from 600 rather than 670 participants were included. Is it right? Would you please make this more clear.*

**Reply: **Thank you for catching this mistake. It’s correct that we analysed only 600 participants’ responses. We’ve clarified this in the revision.

# Reviewer report: Reviewer response for version 1

**DOI:** 10.5256/f1000research.79226.r120530

**Published:** 2022-02-07

**Authors:** Marius Usher

**Affiliations:** 1 School of Psychology, Tel Aviv University, Tel Aviv, Israel

The manuscript examines the amount of information that observers can extract from visual images at very fast rates (67/133/267 ms masked). The researchers present a large set of complex scenes, which observers can not generate expectations about, and rely on a verbal free response method (rather than the traditional forced response) to obtain information about what the observers could extract from the brief and masked display. In order to quantify the accuracy of the responses the authors developed a novel index called Intersubjective Agreement (IA), which reflects the specificity of the responses to a target image. The results indicate that most of the responses show a high IA and correspond to visual details (small object, or letter in a scene), a finding which goes against the traditional wisdom that people can only extract broad (gist or category type) information from such brief displays. Moreover, the IA is correlated with confidence, indicating metacognitive conscious access.

 I find the question that this study addresses an important one and the method innovative and solid. The writing is also quite clear, so I believe this paper could be of interest to the research community. Below I will enter a number of comments, which the authors could address to make their paper more clear and easier to follow, and to explore its implications.

**Methods**

- p. 4. I wonder if the term Word IA, should be Word-Image IA (as it seems to link to a Image-word pair).
- p. 5. "Word-IA are high". Is the point that the values are closed to 1? Is the possible range (0, 1)? It may help to specify this.
- Fig 2c. There should be a legend for the colors here.
- Also it would help to clarify the motivation why one has to rely on such a complex method (ROC curves) for the index, instead of just computing a ration of responses given to the target image over All responses (to all images). Usually ROC curves are used when there is a subjective-criterion difference. It was not very clear why this complex method was needed here. Finally, the construction of Fig 2c may be explained better. (Those cumulative probabilities appears to only have binary 0/1 values…)
- It would help if one could also provide some index of the information that one can extract from the images. (Perhaps this could integrate the IA with the number of words reported…)

**Results**

 There are very interesting results reported here. But to me the most important would be to see how the information extracted from the image (see above) or at least the IA, varies with the presentation rate. While the paper reports that IA are high even at the fastest rate (67 ms), I did not see a Figure showing the dependency on the rate. Finally, it would be quite interesting to know what the temporal limit at which the capacity to extract information from rapid displays breaks down? (I suppose this will need to take place at fast enough rates, of < 20 ms or so). Showing such data will also help to better appreciate the results at 67 ms, as the more rapid presentation would serve as a control, relative to which the present results can be compared.

**Implications**

 Finally, it may be interesting to discuss implications to theoretical debates such as the Overflow in Consciousness research.

Is the work clearly and accurately presented and does it cite the current literature?

Yes

If applicable, is the statistical analysis and its interpretation appropriate?

Yes

Are all the source data underlying the results available to ensure full reproducibility?

Yes

Is the study design appropriate and is the work technically sound?

Yes

Are the conclusions drawn adequately supported by the results?

Yes

Are sufficient details of methods and analysis provided to allow replication by others?

Yes

Reviewer Expertise:

Cognitive psychology

I confirm that I have read this submission and believe that I have an appropriate level of expertise to confirm that it is of an acceptable scientific standard.

# Response

**Published:** 2022-08-21

**Authors:** Zhang Chuyin

**Affiliations:** Monash University, Australia

Thank you for reviewing our paper and providing valuable comments! We will respond your concerns one by one. 

 1, *p.v4. I wonder if the term Word IA, should be Word-Image IA (as it seems to link to a Image-word pair).*

**Reply: **We agree with you that Word-Image IA is more precise and consistent with what we meant by Word IA. However, Word-Image IA is a bit long and the abbreviation of WIIA is not intuitive to grasp. On the other hand, Word IA is concise with a minimal use of abbreviations, IA. Thus, we opted for using Word IA throughout. We’ve added the following to make it clear:

- Footnote: "To be precise, we should perhaps use “Word-Image IA” instead of “Word IA” as it links to an image-word pair. Just for the simplicity and readability, we use “Word IA”."

2, *p. 5. "Word-IA are high". Is the point that the values are closed to 1? Is the possible range (0, 1)? It may help to specify this.*

**Reply: **Yes, the possible range of Word IA is [0, 1]. By “high” we mean the values are close to 1. We’ve clarified this as follows: 

- Page 5: “as we mentioned above, Word IA ranges from 0 to 1. Most words that were reported had very high Word IA (close to 1) across SOAs.”

3, *Fig 2c. There should be a legend for the colors here.*

**Reply: **Thank you. We’ve added in a figure legend for Figure 2c (and newly added e). We also paste the modified explanation of Figure 2 as follows:

-
***See Figure R1 linked here***

- **Figure R1 (Figure 2).** e) Another example for the target word “grass” after viewing (a), whose Word IA = 0.84. They are constructed in the same way as (c) and (d). This example shows values on the red line are not binary.

4, *Also it would help to clarify the motivation why one has to rely on such a complex method (ROC curves) for the index, instead of just computing a ration of responses given to the target image over All responses (to all images). Usually ROC curves are used when there is a subjective-criterion difference. It was not very clear why this complex method was needed here.*

**Reply: **We had empirical and theoretical motivations to introduce the ROC curves instead of a simpler method, like the one you suggested. 

 Empirically, a simpler ratio scale is not easy to interpret. If we define it as: Ratio1 = # of responses given to the target / # of responses to all images (including the target), then, it ranges from 0 to 1. But the distribution is extremely concentrated around 0 and 1 (Figure R2). 

 Theoretically, this method is problematic in that any word that was reported in many images, such as “grass”, can never obtain high value, even if one particular image was reported with “grass” for all of N = 30 people. If “grass” was reported 300 times in total, then this ratio1 index will be 0.1 (= 30/300). Thus, we need some way to normalise the baseline report rate of the word.

**See Figure R2 linked here**

**Figure R2. **Histogram for the distribution of ratio1 (X axis). Ratio1 = [# of responses given to the target / # of responses to all images (including the target)]. We calculated ratio1 for all unique (word, image) pairs.

 This leads to the 2nd type of ratio scheme, Ratio2 =  % of responses given to the target / % of responses to all images (including the target).

 This comes closer to what our ROC is measuring. However, Ratio2 = [X _target_/30]/[X _all_/(420 × 30)] = (X _target_/X _all_) × 420 = Ratio1 × 420. Thus, it can go from 0 to 420 and interpretation of ratio 2 is even harder than ratio 1. We need some alternative methods.

 At first, we wanted to invent an index that is free of subjective criteria, or confidence rating, as you suggested, using ROC. Let us consider “eiffel-tower” in Fig 2a as an example. 13 out of 30 participants reported it under the target image, and 1 participant reported it under other images. Then, our implementation of the ROC goes as follows: 

1. Order the responses according to the confidence ratings to the target image, then to compute a cumulative probability. This serves like a cumulative hit in a standard ROC.  (Figure R3a - green line)
2. Repeat the same procedure as 1) but for all-non target images, which serves like a cumulative false alarm. (Figure R3a - red line)
3. Construct the ROC. (Figure R3b)

**See Figure F3 linked here**

**Figure R3.** a) Cumulative proportion (y-axis) for the target (green) and other images (red). We first order the responses according to the confidence, and count the number of responses for each confidence rating. X axis is confidence rating. If participants did not report “eiffel-tower”, we counted them as 0 confidence rating response, so that cumulative proportion always reaches 1 for both hit (green) and false alarm (red). b) A ROC curve constructed from Figure R3a. X axis is the false alarm rate, which comes from the red line in Figure R3a. Y axis is the hit rate, which comes from the green line in Figure R3a. AUC here is 0.72.

 From the ROC, we computed the area under the ROC, which we call “AUC by confidence”. For the example of “eiffel-tower”, AUC by confidence = 0.72.

 We calculated AUC by confidence for every response word, compared against Word IA in Figure R4a. 

**See Figure R4 linked here**

**Figure R4.** a) The scatterplot showing the relationship between Word IA (x axis) and AUC by confidence (y axis). Red line is the identity.  b) The strong correlation between AUC by confidence and report frequency under the target image. c) Histogram of AUC by confidence (x-axis is the same as panel b). 

 In Figure R3, we showed the cumulative proportions and their corresponding ROC. As we count the proportion of people who did NOT report “eiffel-tower” as confidence = 0, the maximum area under the ROC (regardless of the false alarm) is set by the % of people who reported “eiffel-tower” in the target image, which is 13/30 = 43%. This corresponds to the highest black point in Figure R3b on the y-axis. Although “eiffel-tower” was reported only 1 out of 419 images × 30 responses (nearly 0 false alarm), this means that “eiffel-tower” can attain only up to AUC of 0.72. This means, generally speaking, AUC by confidence can reach 1 when and only when all 30 participants reported the word under the target image. 

 Figure R4a shows a moderate positive correlation between AUC by confidence and Word IA. However, Figure R4b shows an extremely tight correlation between AUC by confidence and the report frequency of the word for the target image. In the case of the “eiffel-tower”, the report frequency of 13 (out of 30) indeed predicts AUC by confidence at 0.72. As a result, their distributions are quite different. AUC by confidence is highly concentrated around 0.55 (Figure R4c). 

 Another feature we see in Figure R4a is that AUC by confidence hardly goes below 0.45. This is because our participants rarely reported the same word across many images. In other words, the baseline report rate of any given word is very low. Among all reported words, “man” was reported most frequently, but it was only around 12% of possible report rate (around 1600 times, out of the possible 420 × 30=12600 times in total under all images). 

 This leads to our proposal of IA. Figure 2c in the main text shows that we use % of participants for cumulative probability to construct ROC. This allows us to characterise a word such as “eiffel-tower” with IA~1.0, which signals that this word is effectively used for this image, but not for the other images. This also explains why in Figure R4a, we see many words with AUC by confidence of ~0.6-0.7 having Word IA of 0.75-1.0. 

 Given the importance of explaining the motivations behind our new IA measure, we decided to include this as a supplementary material. We explicitly mentioned this in the Results section as follows:

- Footnote: "We described our motivations and rationale behind our definition of IA over other simpler methods, such as ratio of reported words for the target vs all images in Supplementary Material (see https://osf.io/nvfhs)."

5, *Finally, the construction of Fig 2c may be explained better. (Those cumulative probabilities appears to only have binary 0/1 values…)*

**Reply: **You are correct in pointing out that both green and red lines in Figure 2c go from 0 to 100%. As long as we do not group multiple images as a target image, the green line always goes from 0 to 100% as it shows the cumulative probability of hit. The red line (other-image) might be different for other examples. We’ve added Figure 2e and 2f (example of “grass”) to show that cumulative possibilities on the red line take values between 0 and 1. This is because the word “grass” was reported in many images with different frequencies (50%, 30%, …). We’ve also added a sentence into the legend of Figure 2c as follows: 

- (Figure 2c) “The value on this curve doesn’t have to be 0 or 1, it jumps from 0 to 1 because no one reported “eiffel-tower” under the other images. ”

6, *It would help if one could also provide some index of the information that one can extract from the images. (Perhaps this could integrate the IA with the number of words reported…)*

**Reply: **We agree with you that it would be great to come up with some index of information that one can extract from a given image. In our results section, we proposed “Image IA” as such an estimate. Image IA is the mean of Word IA under a specific image. 

 Thanks for your suggestion to integrate IA and the number of words reported, which we did not try before. While there can be many ways to integrate them, as an initial attempt, we tested if the sum of the Word IA (called “Sum IA”) per a particular image can work as the desired index. Below, we compare our original Image IA and Sum IA.

 Ideally, the index should be lower for a simple and abstract image, which would be hard to extract specific information and to report many words that are specific for that image. Also, the ideal index should be high if an image contains many distinct, clear and nameable objects.

 Somewhat surprisingly, we found that Image IA and Sum IA are uncorrelated (see Figure R5). But the relationship between them highly depends on the number of unique words reported under an image. In Figure R5, we display 4 examples at the extreme corners (highest/lowest Image IA/Sum IA). 

 By looking at the top left image, we have an impression that high Sum IA (= 26.82) may be related to many words that can describe the image (e.g., rice, wheat, field, farm, yellow, brown, machine etc). However, lower Image IA (= 0.84) may be related to the fact that each word is not very specific to this image and is often reported in other images.

 Now, looking at the bottom right image, it may have a high Image IA (= 0.99) as it is accompanied with very specific words that are not reported in other images (e.g., boat, lake, fast, speedboat, life-vest. See Figure 4a). But as this image does not contain many objects, it may have led to smaller Sum IA (=12.82).

 But it is hard to say why the top right dog image was described with many (Sum IA = 28.6) specific words (Image IA = 0.95) while the wheel image at the bottom left was described with few (Sum IA = 11.7) and less specific words (Image IA = 0.84). 

 Of course, we need to be careful in our inference because our impression here is based on the prolonged and unlimited duration of image viewing. Our IAs are based on the reports by participants who viewed them only briefly (67ms, 133ms or 266ms, masked).

**See Figure R5 linked here**

**Figure R5. **The relationship between Image IA and Sum IA. There is no correlation between Image IA and Sum IA, but the relationship highly depends on the number of unique words reported under an image. Each colour (dots and lines) means a number of unique words reported under an image. Four example images with their Sum IA (below the image) and Image IA (in the bracket) are also shown.

 We also examined other images. Sup Fig 6 and 7 ordered 420 images by their Image IA and Sum IA, respectively ( https://osf.io/m7az6). For the convenience of the reviewer, in Figure R6, we show the 42 images with the highest Sum IA, the 42 images with the lowest Sum IA, and their Image IA. While our impressions above may be largely true, there are also notable exceptions. At this stage, we cannot say which of Image IA and Sum IA captures “the information one can extract” better. Each index is likely to be capturing different aspects of how people experience the scenes at a brief moment and report them in an intersubjectively consistent manner. To say more, we would need more research in the future, which we plan to do. 

**See Figure R6 linked here**

**Figure R6.** a) 42 images with the highest Sum IA, ranked by Sum IA. The Sum IA and Image IA (in the bracket) of each image is shown under the image. b) 42 images with the lowest Sum IA, ranked by Sum IA. The Sum IA and Image IA (in the bracket) of each image is shown under the image.

 7, *There are very interesting results reported here. But to me the most important would be to see how the information extracted from the image (see above) or at least the IA, varies with the presentation rate. While the paper reports that IA are high even at the fastest rate (67 ms), I did not see a Figure showing the dependency on the rate.*

**Reply: **In order to increase the number of observations for each image, we calculated our Image IA (and Sum IA above) across SOA. It means we used N = 30 participants across three SOAs under each image. So the Image IA and Sum IA values above are the same for different SOAs. 

 To address how these values depend on SOAs, we sacrificed the precision and calculated them for each SOA (where we had only N = 10 participants, Figure R7). From the figures we can see that both Sum IA and Image IA at SOA = 67ms are slightly lower than them at SOA = 133 and 267ms (ANOVA: for Image IA, F(2, 809.03) = 71.27, p < .001; for Sum IA, F(2, 836.19) = 41.67, p < .001).

**See Figure R7 linked here**

**Figure R7.** a) The boxplot showing Sum IA per SOA at each SOA. b) The boxplot showing Image IA per SOA at each SOA.

 8, *Finally, it would be quite interesting to know what the temporal limit at which the capacity to extract information from rapid displays breaks down?*

**Reply: **We agree with you that going shorter SOAs will be very interesting. 

 We performed some preliminary experiments with shorter frame rates online. We confirmed its feasibility, but to be confident about the results, we believe that we need to intermix shorter SOAs with longer SOAs and test many participants with many images. Showing images with only shorter SOAs will be feasible to add to our data, but we expect that many participants will start setting a high threshold for reporting when there are no easy trials included (e.g., Lin & Murray 2014 Journal of Vision). Thus, to avoid such an effect, we probably need to replace our 67 ms condition with a shorter condition, while keeping 133 and 266ms conditions. To test the entire image set, this will mean that we need to run another 670 subjects to add the 33 ms (or 17ms) condition. Perhaps, we need to start its feasibility from the in-lab face to face experiment. Overall, we agree that this will be an important direction of research we plan to pursue, but given the time it would take to complete, the length and the density of the current paper, we prefer to do so in the future.

 Reflecting on this, we decided to add the following under “Presentation duration” in Discussion:

- “We performed some preliminary experiments with shorter SOAs online and confirmed its feasibility. To conduct such a study in the future, however, we recommend to confirm the results with in-lab participants to some extent. We also suggest that shorter SOA trials should be intermixed with longer SOA trials, so that participants can set an appropriate threshold for reports (Lin & Murray 2014 Journal of Vision). Such experiments will address an important question on the temporal limit of intersubjectively shared experience upon viewing rapid visual scenes.”

9, *Finally, it may be interesting to discuss implications to theoretical debates such as the Overflow in Consciousness research.*

**Reply: **We agree that our paper has some implications to the theoretical debate on the overflow in consciousness research. Our paper contributes to this debate in two ways. First, is about the Sperling paradigm, which has been the central to the overflow debate. 

 In Sperling’s paradigm (1960), participants are briefly shown an array of 12 letters in 3 rows and 4 columns. Generally, they are believed to report having an impression of seeing all letters. However, under the whole-report condition, where they are asked to report as many letters as they can, they cannot report no more than 4 individual letters accurately. However, when cued on a particular row, after the array disappears, they can report all letters accurately. 

 Our results in Figure 4a cast doubt on this uncontested description of the results in the whole-report condition (See also Figure R6b, Supplementary Material 6 and 7 for two Sperling arrays, with both very high Image IA and Sum IA). Our participants in fact reported highly specific letters ({U, P, L, G, J} in Figure 4a) more frequently than the general impression of “letter array”, “alphabets”. 

 This is consistent with some reviews (e.g., Haun et al., 2017; Bronfman et al. 2019), which propose that participants are actually perceiving more than previously thought. 

 Second, our experiment opens up a possibility to extend the discussion of the overflow debate in more naturalistic situations, while most discussions so far centred around the results obtained with highly artificial stimuli. Nonetheless, our current study has implemented more like the whole-report condition of the Sperling paradigm. By extending it with a pre/post cued partial-report paradigm, we can expect that our paradigm provides a novel methodology to study the overflow debate with a large set of natural scene images. We included these points under “Future directions” in Discussion as follows:

 Page 14: “Finally, our paper has some implications to the theoretical debate on whether conscious experience is restricted by cognitive access or overflows such restriction (Block, 2007 BBS; Bronfman et al., 2019 Mind & Language). Previous debate on the overflow issue was almost always focused on the artificial stimulus situation introduced by Sperling (1960). Our paper contributes to this debate in two fresh ways. First, since the Sperling’s initial introduction of the paradigm, it has been almost always “assumed” that what participants see in the “whole-report” condition is just a gist, such as “seeing everything”, “alphabets”, and “letters” (Haun et al., 2017). Our empirical free-report challenges this notion. Even without any warning or expectation, participants report specific letters rather than generic gists (e.g., Figure 4a). Second, our paradigm opens up a possibility to examine the overflow issue with naturalistic stimuli. For that, we would need to develop equivalent experimental contrasts between the whole- vs. partial reports as in Sperling 1960, which is not impossible."
